# Tolosa-Hunt Syndrome: A Case Report

**DOI:** 10.7759/cureus.84606

**Published:** 2025-05-22

**Authors:** Sarah Alshamlan, Noof Almukhaimar, Laila Bukhamseen, Yasmeen Alnaimi

**Affiliations:** 1 College of Medicine, King Faisal University, Al-Ahsa, SAU; 2 Neurology, Dammam Medical Complex, Dammam, SAU; 3 Radiology, Dammam Medical Complex, Dammam, SAU

**Keywords:** cavernous sinus, diplopia, headache, ophthalmoplegia, tolosa-hunt syndrome

## Abstract

Tolosa-Hunt syndrome (THS) is a rare neurological condition characterized by ophthalmoplegia preceded by retroorbital pain or headache. It mainly involves cranial nerves III, IV, and VI. The majority of the time, the cause is unknown; it is believed that the cause is nonspecific granulomatous inflammation of the cavernous sinus and superior orbital fissure or rarely beyond. THS is an exclusionary diagnosis. It is diagnosed clinically according to the patient's symptoms, neuroimaging test results, and response to corticosteroid therapy. The mainstay of treatment is corticosteroid therapy. A 62-year-old man, a known case of hypertension (HTN) and type 2 diabetes mellitus (T2DM), presented with an acute history of right-sided headache and diplopia, associated with right eye pain and tearing. The patient was stable, with a Glasgow Coma Scale (GCS) score of 15/15. Neurological examination showed right eye ptosis, impairment of lateral and upward movement of the eye, and reduced sensation on the right V1 and V2. The left side of the face was positive for flat nasolabial folds. Later, the patient complained of decreased visual acuity. The diagnosis was confirmed with an orbital magnetic resonance imaging (MRI). The patient improved on prednisolone therapy. THS should be considered in patients presenting with painful ophthalmoplegia, as early diagnosis prevents unnecessary invasive procedures and ensures effective treatment, which can significantly reduce morbidity.

## Introduction

Tolosa-Hunt syndrome (THS) is a rare neurological condition characterized by ophthalmoplegia preceded by retroorbital pain or headache. It mainly involves cranial nerves III, IV, and VI [1]. In 1954, Tolosa created a comprehensive representation of this syndrome. Later, in 1961, Hunt correspondingly reported six cases after conducting additional investigations. Both extraocular nerve palsies and unilateral retroorbital pain have been present in these cases and showed a response to steroid therapy. Approximately one case of THS occurs for every million people annually [2]. THS affects both men and women with no preference, is not age predisposed, and is primarily unilateral [3].

The majority of the time, the cause is unknown. It is believed to be caused by nonspecific granulomatous inflammation of the cavernous sinus and superior orbital fissure or rarely beyond [4]. THS has no known autoimmune cause, but it has been described as the initial symptom of a number of systemic autoimmune inflammatory diseases, including Wegener's granulomatosis, sarcoidosis, and systemic lupus erythematosus [3]. In a study done in Qatar, out of 31 THS patients, only one had a history of an autoimmune disease [5]. In addition, THS has not been linked to any infectious agents. One documented case of THS in a patient with latent tuberculosis was found through a literature search [3].

Even though THS mostly affects cranial nerves III, IV, and VI, in certain documented instances, it can involve multiple cranial nerves, such as the I, VII, or VIII nerves or the V nerve (often the first division), leading to various neurological symptoms. Paresthesia of the forehead may result from involvement of the first division of the V nerve, mainly V1. Loss of the ipsilateral corneal reflex is indicative of involvement of the V nerve. It is also possible to observe mild proptosis or optic disc edema if the orbit is involved. Pupillary dysfunction may arise from inflammatory involvement of the parasympathetic fibers surrounding the III nerve or the sympathetic fibers in the cavernous segment of the internal carotid artery (ICA). Horner syndrome with miosis may result from the involvement of the sympathetic nerves that move through the cavernous sinus's interior [6].

THS is frequently regarded as an exclusionary diagnosis since it lacks a single pathognomonic feature. It is diagnosed clinically by the patient's symptoms, neuroimaging test results, and response to corticosteroid therapy [2]. The International Classification of Headache Disorders (ICHD-3) suggests that the standard diagnostic criteria for THS include experiencing a headache on one side and locating evidence of granulomatous inflammation in the orbit, superior orbital fissure, or cavernous sinus. Magnetic resonance imaging (MRI) or biopsy can be used to verify this evidence. A paresis affecting one or more of the ipsilateral cranial nerves III, IV, and/or VI should also be present. The headache should appear ipsilateral to the area of inflammation and might even occur up to two weeks prior to or concurrent with the occurrence of ophthalmoplegia [7].

Corticosteroid usage is crucial for both diagnosis and therapy. The rapid resolution of pain, typically within 24-72 hours, supports the THS diagnosis, as well as the resolution of the cranial nerve palsies and the reversal of MRI abnormalities within two to eight weeks thereafter [2].

Close follow-up and repeat neuroimaging as necessary are recommended by the ICHD-3 in order to rule out other possible causes of painful ophthalmoplegia, such as tumor, vasculitis, skull base meningitis, sarcoidosis, or diabetes [7].

We reported a new case of THS, describing management and diagnostic investigations undertaken to approach this syndrome. Also, this case underscores the challenge in diagnosing THS and the importance of a multidisciplinary approach toward this kind of condition.

## Case presentation

A 62-year-old Bangladeshi man, a known case of hypertension (HTN) and type 2 diabetes mellitus (T2DM), presented to the emergency room (ER) with a one-day history of right-sided headache and diplopia, associated with right eye pain and tearing. The symptoms started suddenly while the patient was driving the previous day. He indicated that he had a headache history all over the head in the previous five days, which was managed with morphine in the emergency department. 

On clinical examination, his vitals were normal, with a random blood sugar (RBS) of 218. He was conscious, alert, and oriented with a Glasgow Coma Scale (GCS) score of 15/15. His mental status examination was normal. On neurological examination, he had right eye ptosis and impairment of lateral and upward movement of the right eye. There was reduced sensation on the right V1 and V2, but intact V3. The left side of the face was positive for flat nasolabial folds. Motor and cerebellar examinations were unremarkable.

Lab tests with significant results are shown in Table 1. A brain computed tomography (CT) was done and excluded acute injuries. A brain CT angiography was also done and was unremarkable.

**Table 1:** Blood test results. LDL: low-density lipoprotein

Test	Result	Unit	Reference range
Triglycerides	2.46	mmol/L	0-1.7
Cholesterol	6.58	mmol/L	0-5.18
LDL	5.14	mmol/L	0-2.6
HbA1c	7.8	%	4.0-6.0

The patient was admitted under neurology for further investigation and evaluation with a differential diagnosis of brainstem ischemic stroke. He was started on dual antiplatelet therapy (aspirin 81 mg orally (PO), once daily (OD), and clopidogrel 75 mg PO OD) for secondary prevention, alongside atorvastatin. Lab tests for lupus anticoagulant, anti-cardiolipin (IgM, IgG), anti-beta-2 glycoprotein I antibodies, antinuclear antibodies (ANA), antineutrophil cytoplasmic antibody (ANCA) profile (MPO, PR3), anti-Smith antibodies, anti-dsDNA antibodies, anti-SSB (anti-La) antibodies, anti-SSA (anti-Ro) antibodies, anti-Scl-70 antibodies, rheumatoid factor (RF), C3/C4 complement levels, protein C, protein S, factor V, antithrombin III, and angiotensin-converting enzyme (ACE) level were all unremarkable. A brain MRI (fluid-attenuated inversion recovery (FLAIR) and diffusion-weighted imaging (DWI) for stroke) without contrast was performed on the patient, which revealed no evidence of acute infarction with only an impression of small vessel disease. A transthoracic echocardiogram (TTE) was unremarkable.

After ruling out any acute insults, the patient was discharged after three days of hospitalization with home medication consisting of glimepiride 2 mg PO OD, metformin 750 mg PO twice daily (BID), aspirin 81 mg PO OD, clopidogrel 75 mg PO OD (for three weeks only), atorvastatin 40 mg PO OD at bedtime, and valsartan 80 mg PO OD for hypertension. Instructions for reaching the emergency department when necessary were also given, and the patient was scheduled for a brain MRI and outpatient follow-up with neurology. 

One week after discharging the patient, he presented to the ER complaining of decreased visual acuity for one day following his MRI appointment. He described it as complete right eye blindness when looking down, which progressed in severity to where he can only see light. According to the patient, the diplopia persisted until he started to have reduced visual acuity. The right retroorbital pain intensity has improved since discharge but was still present.

On examination, he was vitally stable. The neurological examination was positive for impairment of abduction, adduction, and vertical movement of the right eye. Also, the patient reports increased right retroorbital pain with eye movement. There was right monocular blindness in the visual field. The motor examination was only positive for diminished deep tendon reflexes in the upper limbs bilaterally. Labs showed high erythrocyte sedimentation rate (ESR) and high lactate dehydrogenase (LDH) (Table 2).

**Table 2:** Blood test results. ESR: erythrocyte sedimentation rate; LDH: lactate dehydrogenase

Test	Result	Unit	Reference range
ESR	30	mm/hour	5-10
LDH	314	U/L	120-246

The scheduled MRI brain with contrast images on the previous day showed no acute insults. A CT scan of the brain, along with CT angiography and CT venography, was performed and was unremarkable. An ophthalmological examination was performed. The right eye visual acuity was hand motion. Limitation of abduction, adduction, and elevation of the right eye was observed. Also, a relative afferent pupillary defect was present in the right eye. A suggestion to rule out the involvement of the cavernous sinus was provided.

In order to rule out the involvement of the cavernous sinus, the patient was admitted for further investigation. He was started on anticoagulant (enoxaparin 40 mg subcutaneously (SC) BID) and steroid bolus therapy (methylprednisolone 1 g IV for five days). On the following day, the ophthalmological examination was repeated. The visual acuity was counting fingers at 1 meter. There was a limitation of abduction and adduction of the right eye. Also, the relative afferent pupillary defect was present.

Furthermore, an ear, nose, and throat (ENT) consultation was performed. Due to the impression of a sphenoid sinus infection with intra-orbital extension (most likely mucormycosis given the age and nationality of the patient and the immunocompromised status of the patient, as the patient has T2DM), it was recommended to adjust enoxaparin to 80 mg subcutaneously twice daily and to discontinue methylprednisolone. Liposomal amphotericin was initiated. In addition, the patient also underwent an urgent sinus debridement. A biopsy of the right sphenoid mucosa proved chronic inflammation of the mucosa and ruled out malignancy.

Following up with the patient after five days, he reported improvement in vision but continued to have VI nerve palsy and decreased vision on the right side. Therefore, prednisolone 60 mg PO OD for three days was prescribed and was tapered down by 10 mg every day until discontinued. After one week, he reported more improvement in his vision. Moreover, orbital MRI was planned, with differential THS as the diagnosis.

**Figure 1:**
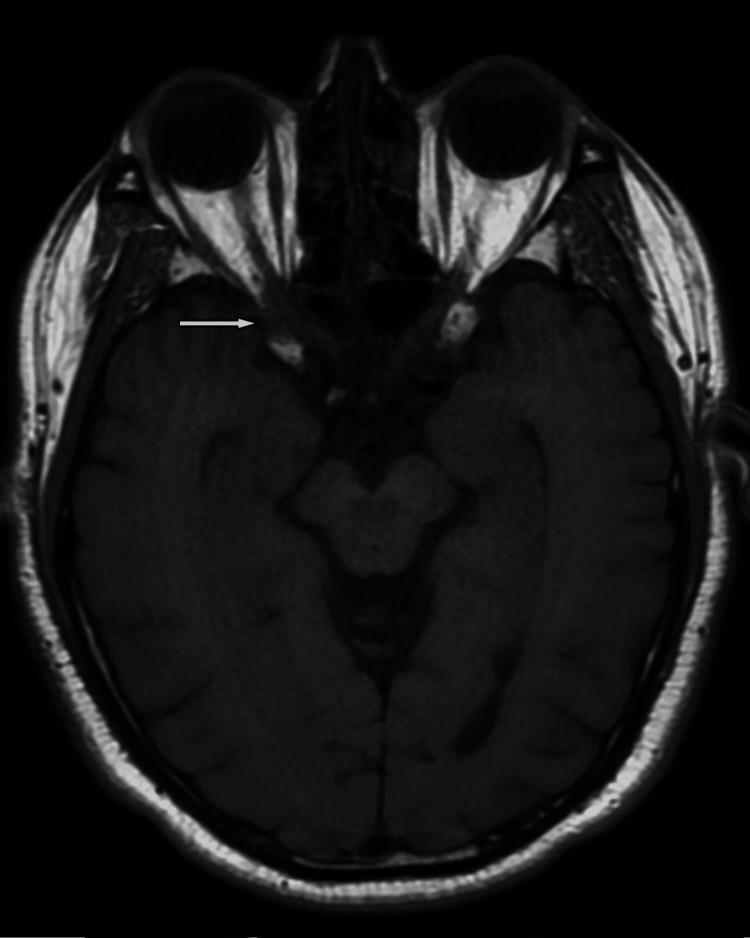
Axial T1 without contrast revealed asymmetry in the cavernous sinuses.

**Figure 2:**
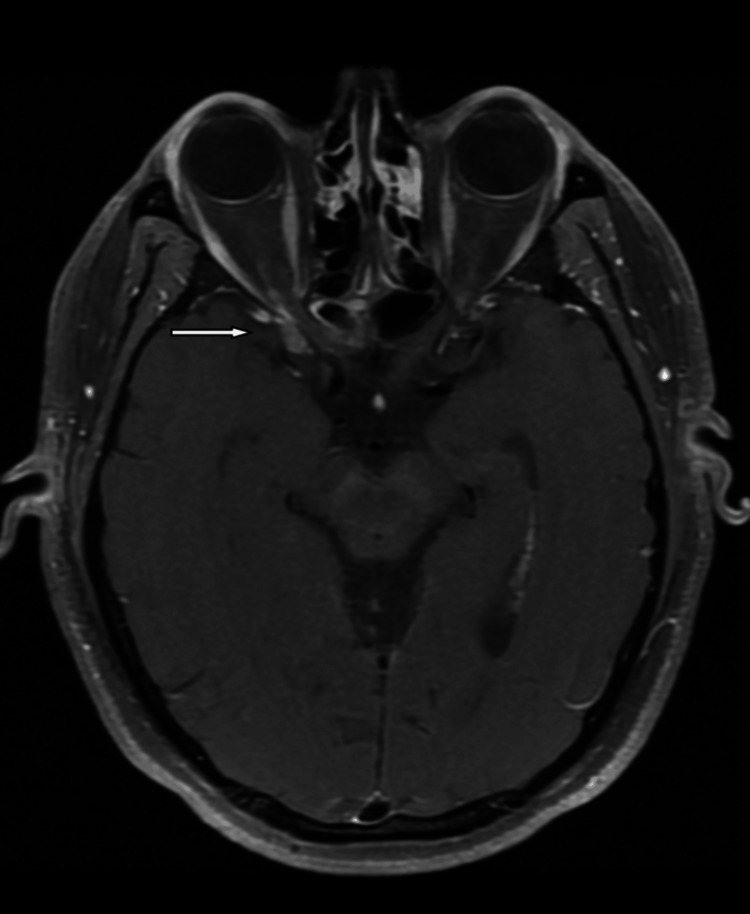
Axial T1 fat-saturated post-contrast revealed asymmetrical increase in enhancement and bulk of the right cavernous sinus extending laterally to the superior orbital fissure and orbital apex. The optic nerve, chiasm, and optic tract are normal in signal intensity with no focal enhancement. Both the orbital globe and ocular and extraocular muscles are unremarkable.

**Figure 3:**
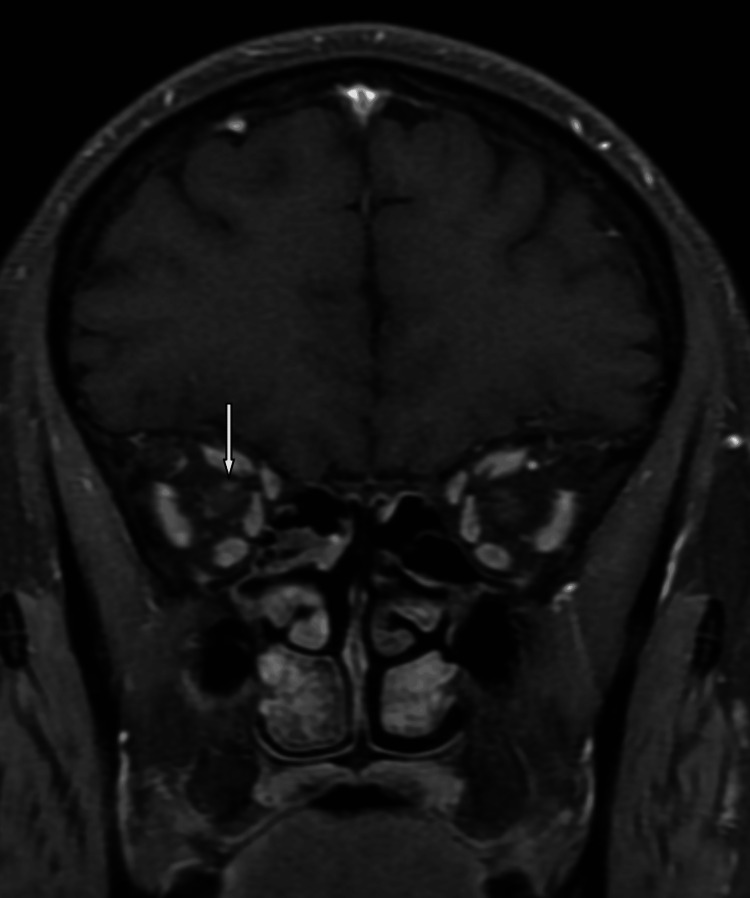
Coronal T1 fat-saturated post-contrast revealed abnormal enhancement of the right optic nerve.

**Figure 4:**
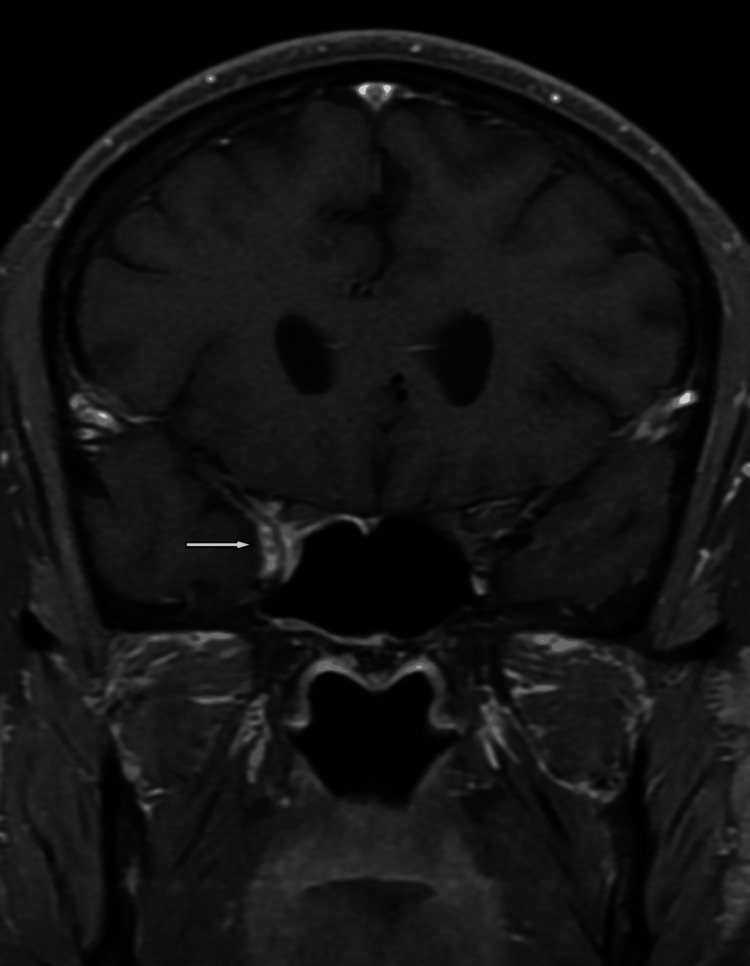
Coronal T1 fat-saturated post-contrast revealed abnormal enhancement of the cavernous sinus.

At the one-month follow-up, the patient improved. Orbital and brain MRI pre- and post-contrast were performed a month later, and the diagnosis of the syndrome was confirmed (Figures 1-4). The patient planned to follow up with ophthalmology.

## Discussion

Our case describes a 62-year-old man who presented with an acute-onset right-sided headache and diplopia, accompanied by right eye pain and tearing. An initial neurological examination revealed impairment of lateral and upward movement of the right eye accompanied by ptosis. About one week after discharging the patient, he started to have decreased visual acuity in the right eye. MRI findings revealed features suggestive of an inflammatory process, while laboratory tests ruled out infectious and autoimmune etiologies. The patient's condition improved on prednisolone. Based on the clinical presentation and the response to corticosteroids, a diagnosis of THS was established and confirmed with orbital MRI, which demonstrated asymmetrical increased enhancement and bulk of the right cavernous sinus extending laterally to the superior orbital fissure and orbital apex.

The first cases of THS were reported by Tolosa and subsequently by Hunt [8]. The precise pathology of the syndrome remains uncertain [4]. It is characterized by unilateral orbital pain and paresis that is caused by granulomatous inflammation in the cavernous sinus, superior orbital fissure, or orbit [2]. 

Depending on the location of the inflammatory process, the patient may present with varying symptoms. The inflammatory process can involve one or more of the ipsilateral cranial nerves that affect ocular motility, such as the III, IV, or VI cranial nerves, or even different combinations of these nerves [2]. In a retrospective study assessing the cranial nerve involvement in THS, the III and IV nerves were the most frequently affected, with 91% for each, while the VI nerve was affected less frequently, by 68% [9]. Furthermore, the ophthalmic branch of the V nerve, as well as the II, VII, and VIII nerves, was also implicated in certain documented cases of THS. Involvement of other nerves, such as the II and VII nerves, suggests that the inflammatory process may extend beyond the cavernous sinus [4]. Madhavan et al. documented an atypical case of THS involving the VII nerve bilaterally [10]. In our case, our patient reported involvement of the II, III, V, and VI cranial nerves. 

Despite the fact that THS is generally acknowledged to be a harmless condition, it is critical to provide immediate evaluation for the patients and to rule out other serious conditions that can present with similar symptoms, such as cavernous sinus thrombosis, malignancies, vascular problems, and other forms of inflammation. Because THS is diagnosed by exclusion, only after ruling out other disorders can a conclusive diagnosis of THS be obtained due to the large number of potential diagnoses and the possibility of misdiagnosis [2]. In this case, due to the patient's risk factors (such as HTN, T2DM, and dyslipidemia) and presenting symptoms, namely, multiple cranial nerve involvements and brain imaging findings (CT, CT angiography, and MRI), that ruled out acute insults, the initial differential diagnosis was brainstem ischemic stroke, which was ruled out with the non-contrast brain MRI (FLAIR and DWI for stroke).

Diagnosis is made by the ICHD-3 diagnostic criteria, which emphasize the presence of unilateral orbital or periorbital headache in less than two weeks or concurrent with cranial nerve impairment (III, IV, or VI cranial nerves), and it should be ipsilateral to the site of granulomatous inflammation evidenced by MRI or biopsy, with no other explanation [7]. Our patient presented with a unilateral orbital headache concurrent with limitation of eye movement at the same site of cavernous sinus involvement demonstrated by MRI.

A contrast-enhanced MRI of the brain is a crucial diagnostic technique, especially when viewed from the coronal aspect [2]. Currently, it is the diagnostic test for THS, as it can confirm the inflammatory etiology of the syndrome, aiding in avoiding invasive tests such as biopsy [11]. Lack of specificity is the main drawback of MRI results in THS [4]. However, it helps rule out a number of disease processes [11]. Yousem et al. reviewed MR images in a retrospective study involving 11 patients diagnosed with THS. Two patients had normal MRI examinations, and nine patients had positive MRI findings. Among these nine patients, eight had extension into the orbital apex. Six of them had an enlarged cavernous sinus. Moreover, five of the nine patients had a convex outer dural margin. One case showed a thrombosed cavernous sinus and superior ophthalmic vein, as well as a soft tissue mass in the cavernous sinus [12]. In this case, MRI revealed increased enhancement and bulk of the right cavernous sinus extending laterally to the superior orbital fissure and orbital apex.

Early therapy can begin as soon as the diagnosis is confirmed. As THS is an inflammatory process, high-dose glucocorticoids are usually used as the first-line treatment. It has been proven that this treatment abruptly resolves orbital pain in 1-3 days, thereby establishing the diagnosis as rapid relief of orbital pain by steroid therapy, which is a characteristic feature of this syndrome [2]. A study done by Zhang et al. found that 40% of patients experienced complete pain relief within 72 hours and the percentage increased after one week to 78% of patients. However, it may take months for neuropathies to resolve, necessitating a longer steroid course [13]. Regarding the appropriate dosage, method, and length of steroid therapy in THS, there are no precise guidelines [3]. Our patient reported a considerable improvement following therapy and full recovery of symptoms within one month.

It is recommended to decrease the oral dosage gradually over a few weeks after an initial high dose of corticosteroids. This should be done with frequent follow-up and MRI scans to evaluate the disease's remission [2]. The resolution of imaging abnormalities should be regarded as "diagnostic" of THS [4].

It has been shown that approximately half of the patients with a THS diagnosis may relapse within the next few months or years following treatment [14]. Recurrence of the syndrome can negatively impact the overall quality of life of the affected people [2]. Recurrence can happen on the same side, on the opposite side, or occasionally on both sides [14]. Neupane et al. reported a case of recurrent THS with more severe symptoms, highlighting the importance of educating patients about re-occurrence and follow-up [15], while Vallinayagam et al. reported a rare case of recurrent THS involving the contralateral eye [16].

In case of suboptimal response to corticosteroid therapy, immunosuppressive medications may be brought into consideration for the treatment [2]. Previous case reports support using immunosuppressive medications such as infliximab and azathioprine as alternatives to corticosteroids in treating cases of THS where patients show poor response or corticosteroids are insufficient or cause side effects [17,18].

One of the main difficulties in diagnosing THS is the broad differential diagnosis, which makes early diagnosis difficult. In addition, THS can mimic more serious conditions, such as neoplasms and vascular conditions. It highlights the need for detailed history, careful clinical assessment, and imaging to exclude the alternative conditions, for instance, cavernous sinus thrombosis, vasculitis, neoplasms, and infections, as well as to avoid unwarranted invasive procedures. The absence of definitive laboratory tests confirms THS, emphasizing the importance of correlating clinical presentation with imaging findings, as the diagnosis of THS is mainly clinical.

Our patient underwent multiple evaluations with different specialties before being diagnosed with THS, highlighting the challenge in diagnosing THS due to its rarity and the nature of its presentation with nonspecific and overlapping symptoms. Additionally, it highlights the importance of a multidisciplinary approach to accurately diagnose complex and rare conditions.

## Conclusions

This is a case of THS presenting with right-sided headache, eye pain, and diplopia, accompanied by ophthalmoplegia and tearing. Diagnosis was confirmed by pre- and post-contrast MRI of the orbits and brain after the exclusion of other alternative differential diagnoses. The patient responded to corticosteroid therapy. This case emphasizes the importance of a systematic, multidisciplinary approach to accurately diagnose and effectively manage THS and exclude other alternative conditions with overlapping symptoms. It is key to understanding that THS should be considered in patients presenting with painful ophthalmoplegia, as early diagnosis prevents unnecessary invasive procedures and ensures effective treatment, which can significantly reduce morbidity.
